# Recent Advances on SEM-Based *In Situ* Multiphysical Characterization of Nanomaterials

**DOI:** 10.1155/2021/4426254

**Published:** 2021-06-09

**Authors:** Juntian Qu, Xinyu Liu

**Affiliations:** ^1^State Key Laboratory of Tribology & Institute of Manufacturing Engineering, Department of Mechanical Engineering, Tsinghua University, Beijing 100084, China; ^2^Beijing Key Laboratory of Precision/Ultra-Precision Manufacturing Equipments and Control, Tsinghua University, Beijing 100084, China; ^3^Department of Mechanical Engineering, McGill University, Montreal, H3A 0G4, Canada; ^4^Department of Mechanical and Industrial Engineering, University of Toronto, Toronto, M5S 3G8, Canada

## Abstract

Functional nanomaterials possess exceptional mechanical, electrical, and optical properties which have significantly benefited their diverse applications to a variety of scientific and engineering problems. In order to fully understand their characteristics and further guide their synthesis and device application, the multiphysical properties of these nanomaterials need to be characterized accurately and efficiently. Among various experimental tools for nanomaterial characterization, scanning electron microscopy- (SEM-) based platforms provide merits of high imaging resolution, accuracy and stability, well-controlled testing conditions, and the compatibility with other high-resolution material characterization techniques (e.g., atomic force microscopy), thus, various SEM-enabled techniques have been well developed for characterizing the multiphysical properties of nanomaterials. In this review, we summarize existing SEM-based platforms for nanomaterial multiphysical (mechanical, electrical, and electromechanical) *in situ* characterization, outline critical experimental challenges for nanomaterial optical characterization in SEM, and discuss potential demands of the SEM-based platforms to characterizing multiphysical properties of the nanomaterials.

## 1. Introduction

The last two decades have witnessed the extensive research on nanomaterials because of their exceptional promise in science and technology. Based on structural dimension, existing nanomaterials fall into four categories of nanostructures: zero-dimensional structures (e.g., nanoparticles, nanospheres, and isolated molecules) [1], one-dimensional structures (e.g., nanowires, nanobelts, nanotubes, and nanoribbons) [2–4], two-dimensional structures (e.g., nanofilms, grapheme, and molybdenum disulfide) [5, 6], and three-dimensional structures (e.g., nanocombs, nanoflowers, and nanocups) [7–9]. Due to their superior physical properties and unique nanoscale morphologies, these nanomaterials have been widely used for a variety of applications such as next-generation electronics [10], sustainable energy [11], biosensing [12], and (opto) electronics [13]. The mechanical, electrical, and optical properties of these nanomaterials play critical roles in their practical uses, and the experimental determination of these properties is thus of major concern from the perspective of both nanomaterial synthesis and applications.

Among various experimental techniques employed for nanomaterial characterization, emerging technique of nanorobotic manipulation in scanning electron microscopy (SEM) has enabled various multiterminal characterization of nanomaterials and nanostructures, such as electrical and mechanical measurements [14, 15]. On one hand, nanomanipulation has filled the gap between top-down and bottom-up approaches and realized position control at the nanometer scale [16] and provides effective strategy for the property characterization of individual nanoscale materials and the construction of nanoscale devices [16]. On the other hand, SEM can provide real-time imaging with nanometer resolution and a large scanning area, which enables the development and integration of robotic nanomanipulation systems inside large vacuum chamber to realize simultaneous imaging and direct interactions with objects at the submicrometer and nanometer scales [14]. In addition, SEM can also be integrated with the latest technology (e.g., electron beam lithography (EBL) and focused ion beam (FIB)) to perform *in situ* nanomaterial engineering and fabrication [17].

Benefiting from above merits, the combination of nanomanipulation technique and SEM has extended both our eyes and hands simultaneously to nanoscale providing an intuitionistic, real-time, and *in situ* way to study nanomaterials and perform nanomaterial characterization in SEM [15]. However, due to existing challenges in the optics integration into SEM, most nanomaterial *in situ* characterization techniques in SEM are limited in mechanical, electrical, and electromechanical measurements; few work has been reported for optical characterization of nanomaterials.

This review presents a survey of recent advances in *in situ* multiphysical characterization of nanomaterials in SEM, including mechanical, electrical, and electromechanical characterization. Challenges and limitations of the optical characterization in SEM are analyzed, and prospects for multiphysical nanomaterial characterization are also discussed.

## 2. *In Situ* Multiphysical Characterization in SEM

Regarding the topic of SEM-based nanomaterial characterization, there are some several reviews in the literature. Fukuda et al. [18] reviewed the assembly of nanodevice and the *in situ* property characterization of carbon nanotubes through nanorobotic manipulation. Shi et al. [14] also reviewed the applications of nanorobotic manipulation in the characterization of nanomaterials and nanostructures. Haque et al. [19] and Zhu et al. [20] reviewed the recent advances in MEMS-based devices for nanomechanical characterization. Besides, Fukuda et al. [21] and Shen et al. [22] reviewed the advanced applications of micronanorobotic manipulation on single-cell analysis and characterization in ESEM. Jiang et al. [23] reviewed the recent advances on *in situ* SEM mechanical and electrical characterization of low-dimensional nanomaterials; the common electromechanical characterization methods of piezoelectric one-dimensional materials were reviewed by Majid et al. [24] as well.

In the following sections, we will focus our review on the topic of *in situ* multiphysical characterization of nanomaterials in SEM, including mechanical, electrical, and electromechanical fields-coupled characterization. Different from the previous review [14] focusing on nanorobotic manipulation systems, we will mainly discuss the characterization methodologies based on different testing types (experimental setups) in each field. In the meanwhile, we will analyze the current status of optical-measurement-related nanomaterial characterization in SEM.

### 2.1. Mechanical Characterization in SEM

The understanding of mechanical properties of nanomaterials plays important roles in miniaturized electronic, optical, thermal, and electromechanical systems. However, due to the scaling effects and geometric differences, when the surface-to-volume ratio increases along with the decreased size of structures, nanostructures such as nanowires (NWs), carbon nanotubes (CNTs), and ultrathin films tend to exhibit significantly different mechanical properties compared with their bulk counterparts [25–27], which means that we cannot easily deduce nanomaterial mechanical properties from bulk properties. Besides, the well-established techniques for mechanical characterization at macroscale cannot be totally transplanted to nanoscale in the respect of equipment and resolution limitations [28]. SEM-based nanomanipulation deals with above challenges for mechanical characterization of nanomaterials in various ways, which are summarized and classified by the testing type (Table 1).

#### 2.1.1. SEM-Based *In Situ* Mechanical Characterization

**Figure 1:**
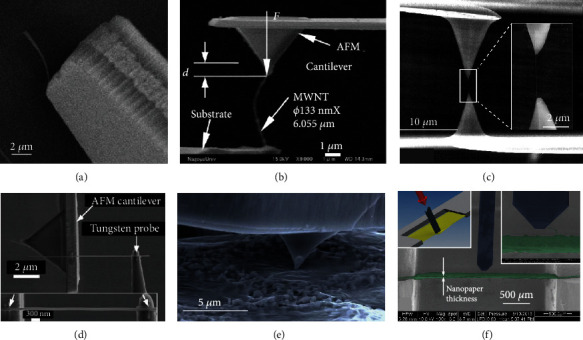
SEM-based *in situ* mechanical characterization of nanomaterials. (a) Deflection of an individual multiwalled carbon nanotube (MWCNT) using a piezoresistive atomic force microscope (AFM). (b) *In situ* mechanical characterization of a nanotube by buckling test. (c) Tensile test of individual MCNT, inset of (c) shows the enlarged view of MCNT under tensile force. (d) Tensile test of a single Ag NW, inset of (d) shows a high-resolution SEM image of the NW for strain measurement. (e) The AFM probe is deflecting the graphene flake while measuring the acting forces. (f) Measurement scene of nanopaper inside SEM: the top left inset shows the stretched nanopaper, and the top right inset shows the nanopaper thickness measurement.

*In situ* bending test was performed on individual multiwalled carbon nanotubes (MCNTs) to characterize its Young's modulus, where the bending force was detected by a piezoresistive atomic force microscope (AFM) probe [29], as shown in Figure 1(a). Also, the individual MCNT's Young's modulus can be determined by *in situ* buckling test [18, 30], as shown in Figure 1(b). An individual MCNT was EBID-fixed with AFM cantilever probe via nanomanipulation, and the buckling force was measured by the deflection of cantilever beam [18].

*In situ* tensile test [31] was employed to study the strength and breaking mechanism of MCNTs, as shown in Figure 1(c). An individual MCNT was EBID-mounted between two opposing AFM tips with different cantilever stiffness, the upper rigid cantilever was driven upward to apply tensile load to the MCNT, and the tensile force was determined by tracking the deflection of the lower soft cantilever [31]. With a similar principle, mechanical characterization of InGaAs/GaAs nanosprings [32], Si nanowires [33, 34], and Ag nanowires [35] was also performed by *in situ* tensile tests, as shown in Figure 1(d). Accurate strain measurements based on high-resolution SEM imaging of Ag NWs facilitated the acquisition of full spectrum of mechanical properties including Young's modulus, yield strength, and ultimate tensile strength [35].

Besides above testing methods, for fragile two-dimensional materials, as a nondestructive assessment method, *in situ* nanoindentation measurements have been employed to examine the mechanical properties of a few-layer graphene membrane [36], individual graphene flakes [37] (Figure 1(e)), and nanopaper made of microfibrillated cellulose [38] (Figure 1(f)), where characteristic force-displacement curves were recorded during the indentation process to extract the Young's modulus. Also, local stiffness of InP suspended micromembrane was first-time measured by a tuning-fork-based dynamic force sensor inside SEM [39]. The surface mechanical properties of low-density polyethylene (LDPE) reinforced by carbon nanofibers (CNFs) were investigated using nanoindentation as well [40].

Except for the abovementioned cases where mechanical properties were *in situ* characterized in SEM vacuum chamber, there are also certain scenarios where SEM was employed as critical tool for surface morphology imaging, which could assist the following mechanical characterization of nanomaterials/nanostructures. Gantayat et al. [41] wrote a review on carbon nanomaterial-reinforced epoxy composites; based on the summary in this review, SEM was proved to be an extremely useful tool to investigate the microstructure and surface morphology of many nanomaterials; moreover, SEM-based results could provide important information for correlated mechanical characterization process [42]. Specifically, the evaluation of surface modification impact on PP/MWCNT nanocomposites by mechanical characterization was assisted with SEM morphological image processing [43]. Similarly, the surface micrographs of nanocomposite films were procured by SEM to explore the nanoparticles' dispensation [44], assisting the demonstration of greater mechanical properties (high tensile strength and Young's modulus). What is more, SEM was also used to characterize the phase separation process in thin electrical conductive composite (ECC) films mixed with nanostructured polyaniline (PANI) [45], proving its enhanced mechanical durability.

#### 2.1.2. ESEM-Based *In Situ* Mechanical Characterization of Biological Cells

**Figure 2:**
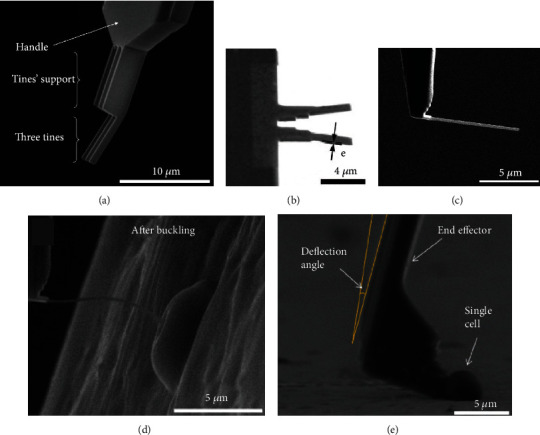
ESEM-based mechanical characterization of biological cells. SEM images of (a) nanofork, (b) nanopicker tip, and (c) soft buckling nanoneedle. (d) Single cell global stiffness measurement using Si nanoneedle in buckling condition. (e) Cell adhesion force measurement.

For cell mechanical characterization in Environmental SEM (ESEM), standard AFM cantilevers were modified using FIB etching and deposition to produce different types of functional tools [14], such as nanofork [46], nanopicker [48], soft buckling nanoneedles [51–55], and flat AFM cantilever tips [49, 50], as shown in Figures 2(a)–2(c). These customized end-effectors were mounted onto the nanomanipulation system in ESEM for indentation to *in situ* characterize the stiffness [51, 52, 55] and viscoelastic properties [53] of single cells (Figure 2(d)), as well as the mechanical properties of individual yeast cells [49] and cell nucleus [54].

Besides, cell-surface adhesion force is important for cell activities and the development of biomaterials, and an *in situ* cell force measurement system was developed based on nanorobotic manipulation inside an ESEM (Figure 2(e)) to characterize the single cell adhesion force [46, 47], cell-surface adhesion force [50], and cell-cell adhesion force [48]. Shen et al. [56] also developed a dynamic force characterization system to investigate the cell detachment process at small scales.

#### 2.1.3. MEMS-Based *In Situ* Mechanical Characterization in SEM

The MEMS-based tensile testing has been applied to characterizing 1D nanostructures (e.g., nanowires, nanotubes, and nanoribbons) and can measure the sample's mechanical properties such as Young's modulus, failure strain and fracture strength [57]. In a tensile test, a 1D nanosample is mounted across a micrometer-sized gap on a MEMS device, and an on-chip microactuator stretches the sample from one side of the gap, and a microforce sensor measures the tension force of the sample on the other side of the gap. The elongation (thus tension strain) of the sample can be quantified via high-resolution imaging (using an optical or electron microscope) [58–60] or on-chip measurement of the sample-mounting gap size [61].

Various typical MEMS platforms were developed for tensile test in SEM, taking advantage of its high-resolution real-time imaging capability for *in situ* observation of material behaviors. Tracing back to the year of 2001, Haque and Saif [62] have proposed the potential application of MEMS actuators on micromechanical testing in an SEM chamber, based on a demonstration of uniaxial tensile test on freestanding thin films in the microsubmicrometer-scale using MEMS devices. The fact of very small overall setup size facilitates the *in situ* observation of materials behavior in SEM chamber [62]. Zhu et al. [59] reported the development of a material testing system for *in situ* electron microscopy (EM) mechanical testing of nanostructures and demonstrated *in situ* EM testing of free-standing polysilicon films, metallic nanowires, and carbon nanotubes. Espinosa et al. [63] developed the first MEMS-based material testing scheme that can continuously observe specimen deformation with subnanometer resolution and simultaneously measure tension force with nano-Newton resolution. B. Pant et al. [58] proposed a versatile MEMS material testing setup that supports both *in situ* and *ex situ* testing of nanomaterial with high accuracy and precision. Except for the abovementioned work, there are also MEMS material testing systems for characterizing 2D nanoscale films [64, 65] and 1D nanomaterials [66, 67].

**Figure 3:**
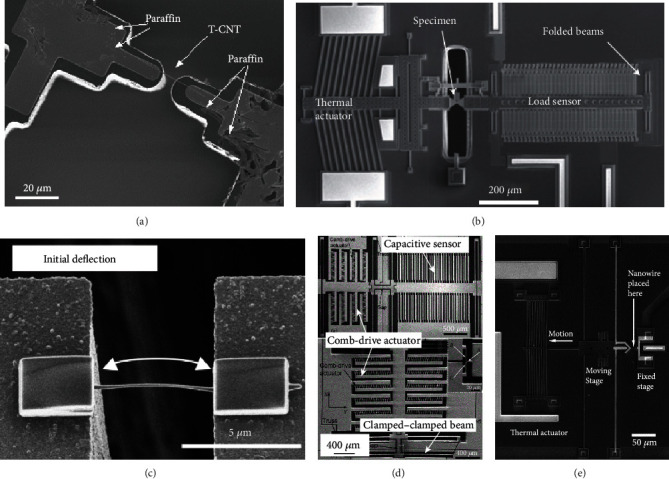
MEMS-based *in situ* mechanical tensile characterization of nanomaterials in SEM. (a) *In situ* tensile loading of a templated carbon nanotube (T-CNT). (b) *In situ* MEMS tensile tester for mechanical characterization of multiwalled nanotubes (MCNTs). (c) FE-SEM micrograph of a carbon nanowire with initial deflection. (d) Two types of nanotensile stages: top picture shows stage with capacitive sensor and bottom one with a clamped-clamped beam force sensor. (e) Microfabricated tensile test structure consisting of electrically isolated moving and fixed stages.

Among various types of nanomaterials *in situ* characterized by MEMS-based tensile platforms, carbon nanotube (CNT) is the representative one. The mechanical properties of CNT were characterized by various *in situ* MEMS platforms in SEM: as shown in Figure 3(a), *in situ* tensile loading of a templated carbon nanotube (T-CNT) was reported in [68], where the load was derived from the bending of the direct force-sensing beam and the elongation of the specimen can be obtained from SEM images. Peng et al. [69] employed *in situ* MEMS tensile tester and exploited the excellent mechanical properties of CNTs with extreme high fracture strength. As shown in Figures 3(b) and 3(a), multiwalled nanotube was bridged between the gap of the actuator (left) and the load sensor. Also, the *in situ* mechanical characterization of free-standing cofabricated polysilicon films and multiwalled carbon nanotubes (MCNTs) in SEM was employed as a validation MEMS-based material tensile testing system, which for the first time achieved continuous observation of the specimen deformation and load measurement electronically with nano-Newton resolution [63]. Zhu et al. [59, 70] designed a MEMS device for the tensile testing of CNTs with two types of actuators: thermal and electrostatic actuators. The device with a thermal actuator [59] was used for displacement-controlled testing and the one with a comb-drive electrostatic actuator [70] for force-controlled testing.

Except for CNTs, the mechanical properties of a series of nanowires have also been characterized by *in situ* MEMS platforms in SEM. Kiuchi et al. [67] employed electrostatic actuated nanotensile testing devices (EANATs) to measure the mechanical properties of single carbon nanowire which is suspended between actuation beams (Figure 3(c)), and the Young's modulus and fracture stress-strain of the nanowires were accurately obtained. Zhang et al. [71] performed uniaxial quasi-static tensile testing on individual nanocrystalline Co NWs in SEM using on-chip MEMS tensile testing system consisting of a comb-drive actuator and a clamped-clamped beam force sensor. As an extended work, Zhang et al. [72] further developed two types of electrostatically actuated tensile stages with either a differential capacitive sensor or a clamped-clamped beam force sensor for mechanical characterization of individual Si NWs, as shown in Figure 3(d). Besides, the fracture mechanism of zinc oxide nanowires was investigated under uniaxial tensile loading utilizing a MEMS-based nanoscale material testing stage [73]. Employing thermal actuator, Brown et al. [74] reported direct tensile tests on n-type (Si-doped) gallium nitride single crystal nanowires (GaN NWs), as shown in Figure 3(e), where tensile strength of NWs was characterized and the failure modes were analyzed.

Similar to tensile tests, mechanical properties of nanomaterials can also be evaluated via MEMS-based bend testing, which represents another type of widely used experimental technique [78]. In a typical MEMS-based bend testing setup [78], a cantilever beam (cofabricated with the MEMS device) is moved by a comb-drive electrostatic actuator and bent against a fixed block. Haque and Saif [62] proposed a MEMS-based setup, which employs a comb-drive electrostatic actuator to bend test a 100 nm thick aluminum cantilever beam. The applied force was calculated using the precalibrated loading equation of the actuator, and the beam deformation was measured via high-resolution imaging. Corigliano et al. [77] proposed a rotary comb-drive actuator and a parallel-plate electrostatic actuator for in-plane and out-of-plane bend testing of thin-film (700 nm) polysilicon microstructures, respectively. The microstructures were cofabricated via a commercial surface micromachining process, which allows for nanometer-thick polysilicon structures to be attached to the bottom of micrometer-think polysilicon MEMS structures. This cofabrication process eliminates the need for nanosample addition after MEMS device fabrication (which could be technically challenging).

Based on above descriptions and discussions, the MEMS-based mechanical characterization techniques of micro- and nanomaterials are summarized in Table 2, classified by different testing types and different categories of materials.

### 2.2. Electrical Characterization in SEM

Better understanding of the electrical properties of nanomaterials will contribute to the development of next-generation nanoelectronics and nanosensors which promise ultrahigh performance [79]. Typically, there are three kinds of methods adopted during *in situ* electrical characterization of nanomaterials: four-point, two-point, and three-point probing.

#### 2.2.1. Four-Point Probing

Four-point measurement is a widely adopted technique to eliminate the effect of contact resistance and has been used for quantifying electrical properties of various nanomaterials such as metallic nanowires [80] and carbon nanotubes [81].

**Figure 4:**
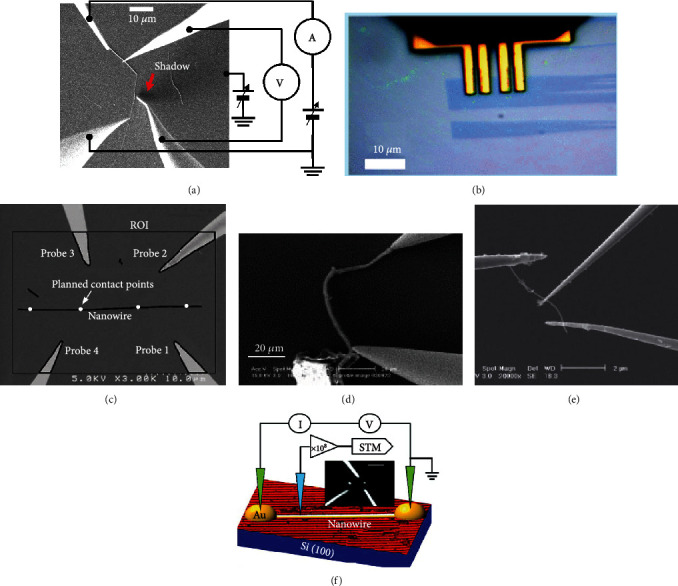
SEM-based *in situ* electrical characterization of nanomaterials. (a) Four-point electrical transport measurement of single CNT. (b) Four-point probing of graphene flake. (c) Visual servo automated four-point probing of single nanowire. (d) Two-point electrical measurement of single nanotube by using two nanoprobes. (e) Three-point field effect measurements on CNT. (f) Three-point measurement configurations for *in situ* electrical transport and local density of states on a single GdSi_2_ nanowire.

Similar with *in situ* mechanical characterization, CNTs were also frequently adopted as the testing material in the SEM-based *in situ* electrical characterization. Four-point electrical transport study of single CNT [82] was performed by a combinatory low temperature four-probe scanning tunneling microscope (STM) and SEM, as shown in Figure 4(a). A reliable nanorobotic system consisting of electrothermal microgrippers and mobile microrobots for automated handling and electrical characterization of CNTs was reported in [83].

Except for CNTs, a series of 2D nanomaterials and nanowires were also electrically characterized using four-point probing technique. To achieve rapid prototyping of graphene-based devices, a nanorobotic platform was developed for time-saving electrical characterization of graphene [84], where four-point probing of graphene flake was shown in Figure 4(b). Similarly, a four-point probe measurement of individual SnO_2_ nanowires was achieved by visual servo automated nanomanipulation inside SEM [85], as shown in Figure 4(c).

#### 2.2.2. Two-Point Probing

Compared with four-point probing, there are still experimental scenarios in which four-point probing is less feasible for electrical characterization of nanomaterials. For instance, certain types of nanomaterials (e.g., III-nitride nanorods) have relatively low aspect ratios, making it difficult to establish four-point contacts along the sample length. Additionally, to characterize as-grown nanowires vertically attached to their growth substrate, it is more convenient to conduct *in situ* two-point nanoprobing, with one probe on top of a nanowire and the other on the growth substrate [86].

For CNTs-related two-point electrical characterization, back to 2004, Peng et al. [87] reported a four nanoprobe system in SEM for two-point current-voltage (I-V) measurement of carbon nanotube, as shown in Figure 4(d). Chen et al. [88] obtained linear I-V measurement curves on MCNTs by establishing two-point Ohmic contacts on a CNT using the Joule heating effect.

A bottom-up technique for nanomanipulation combining STM and SEM was proposed in [89]; the author fabricated two nanocontacts at the end of the GdSi_2_ nanowire and performed direct electrical transport measurement. A metal-semiconductor-metal (M-S-M) model for quantitative analysis of current-voltage characteristics of semiconducting nanowires is proposed in [90], and two-terminal probing was employed for experimental I-V characterization of Bi_2_S_3_ nanowire transistor. Besides above 1D nanomaterials, the electrical conductivity of promising 2D MXene nanosheets was demonstrated by the two-point probing method [91].

#### 2.2.3. Three-Point Probing

Three-point field effect measurements were carried out on CNT by Chen et al. [88] using three-point probing technique where a third probe employed as the gate pole, illustrated in Figure 4(e). *In situ* three-point electrical nanotransport measurements of individual GdSi_2_ nanowires were carried out to investigate the electrical conductance property, where Au-coated STM tip was employed as the third probe [92], as shown in Figure 4(f). Similarly, to evaluate the electrical characteristics of the isolated Nb_2_Se_9_ flake, electrical transport measurement of single Nb_2_Se_9_ field effect transistor (FET) was conducted using Si tips coated by Cr-Pt [93].

Besides above three types of SEM-based *in situ* electrical characterization, there are also certain scenarios where SEM was employed as important tool in assisting the probe of advanced electrical properties of nanomaterials. Specifically, taking advantage of electrons' interaction with the sample atoms in SEM, Mayeen et al. [97] investigated the underlying information about the sample's electrical conductivity. To thoroughly understand the reason why such a small amount of nanostructured polyaniline (PANIs) can tremendously enhance the electrical conductivity of the ECCs, SEM was used to characterize the phase separation process and the self-assembly process in electrical conductive composite (ECC) films [45]. In a most recent work [98], a high-throughput electrical characterization of nanotechnology device arrays was enabled by SEM techniques in selection of purely single nanowires. SEM-based *in situ* characterization techniques could also be applied to tracking the structural reconstruction of the catalysts; in a recent perspective, Zhu et al. [99] established an “*in-situ* probing map” and offered guidelines for the successful development of next-generation efficient electrocatalysts.

#### 2.2.4. ESEM-Based *In Situ* Electrical Characterization of Biological Cells

The electrical characterization of single cells is challenging cause deep penetration of nanoprobe into the cells will burst it with high stress level risk [21]; this issue was solved by performing short penetration of dual nanoprobe, and the single cell's electrical conductivity was measured [94]. Besides, for the first time, the electrical property of single cells under native condition was reported in [95], where single pulse current measurement was carried out on single cells using dual nanoprobe via a ESEM nanomanipulator system. Also, the electrical response of the human embryonic kidney cell corresponding to external mechanic stimulation was studied by Zhang [96] based on a robot-assisted AFM manipulation system. However, there are still some challenges for electrical characterization of the single cell electrical conductivity, such as limited throughput and sensing ability of robotic manipulation system [22].

Based on above reviews and discussions, the electrical characterization of nanomaterials in SEM is summarized in Table 3, classified by different testing types, categories of nanomaterials, and different kinds of end-effectors.

### 2.3. Electromechanical Characterization in SEM

In addition, the intrinsically coupled electromechanical properties of nanomaterials such as piezo-electrical [100–102] and piezoresistive [103, 104] properties have provided special routes of detecting mechanical loading from the electrical change of the nanomaterial and controlling mechanical deformation of nanomaterials via electrical excitation. In the meanwhile, the electromechanical characterization of thin films, nanowires, and nanobelts benefits their potential applications in biosensor development [100, 105], actuators, and motion-controllers [78]. Therefore, it is of great interest to carry out dual-field electromechanical characterization of nanomaterials [106, 107].

#### 2.3.1. Electromechanical Characterization of CNT

Tracking back to the year of 1999, there has been reported work [108] exploring the correlation between mechanical and electrical properties of carbon nanotubes, where carbon nanotubes were stressed while monitoring their conductivity under real-time SEM inspection. Subsequently, the electromechanical characterization of carbon nanotubes [15, 109, 110] was carried out to investigate the coupling effect between its mechanical and electrical properties, such as the resistance SEM *in situ* measurement of CNT versus the stress/strain property [109] and the electrical properties of various types of suspended single-walled CNTs under the influence of tensile stretching [110], as well as the effects of axial strain on electrical transport properties of individual thin CNTs [15].

#### 2.3.2. Piezoresistance Effect Investigation

**Figure 5:**
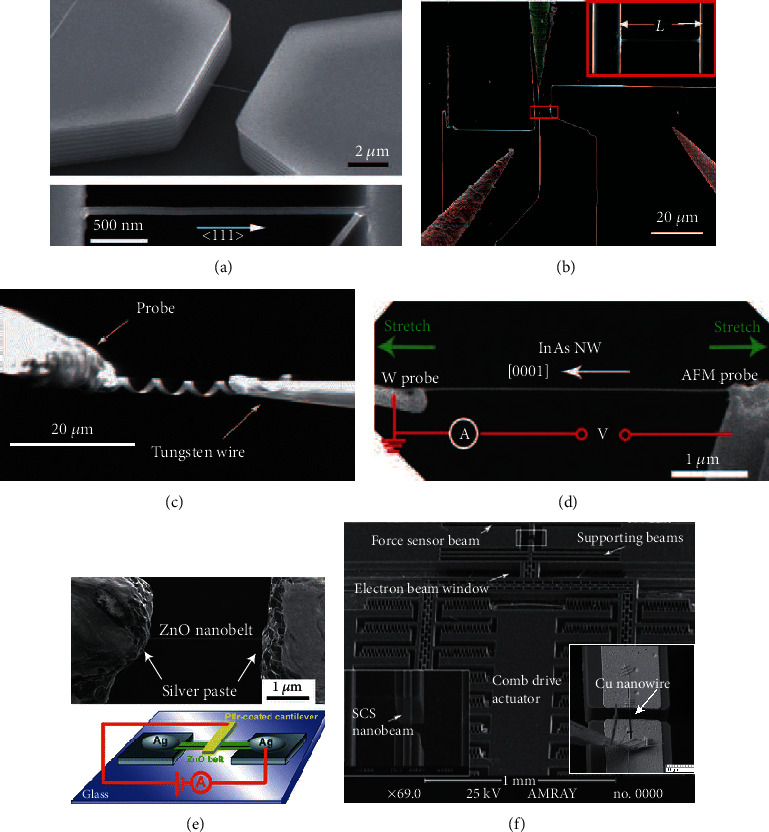
Electromechanical characterization of nanomaterials. (a) Giant piezoresistance effect: single Si nanowires bridged in the trench of SOI substrate. (b) MEMS loading system for tensile testing of a suspended Si nanowire and measuring the resistance change simultaneously. (c) Conductivity measurement of deformed InGaAs/GaAs nanosprings as electromechanical sensor. (d) Electromechanical measurement of an InAs NW using a W probe and an AFM probe, the NW is uniaxially stretched, and its electrical transport properties are measured at different tensile strains. (e) SEM image of a single Sb-doped ZnO nanobelt fixed by using the silver paste and the schematic diagram of the electromechanical characterization measurements. (f) SEM image of MEMS tensile-testing chip, Cu nanowire is integrated onto the MEMS nanobeam, and its mechanical and electronic properties under different stress were characterized simultaneously.

The piezoresistance effect of silicon nanowires has also been widely investigated [111] in order to improve the performance of silicon transistors. For the first time, the giant piezoresistance effect in Si nanowires (as shown in Figure 5(a)) was discovered in [111], which predicted significant perspective in nanowire-based flexible electronics and NEMS. The phenomenon of giant piezoresistance in silicon NWs was further well controlled in [112] for potential application of stress-gated field-effect transistor with a high gauge factor. Anomalous piezoresistance effect [113] was discovered for p-type single crystal silicon nanowires under ultrahigh strain, as illustrated in the SEM image in Figure 5(b). A Si nanowire was suspended in the microelectromechanical testing module. To avoid the effect of electron beam (e-beam) irradiation during nanomaterial testing, Zhang et al. [79] developed a MEMS device for piezoresistivity characterization of synthetic silicon nanowires, where simultaneous electrical and mechanical characterization of individual Si nanowires could be carried out.

The coupled piezoresistive characterization of some other types of nanostructures has also been carried out through nanomanipulation and electron-beam-induced deposition (EBID) inside SEM. For instance, as shown in Figure 5(c), the conductivity of the deformed nanospring [114] was investigated experimentally; the electromechanical properties of InGaAs/GaAs nanosprings were also characterized in [115], illustrating a potential way to realize electromechanical sensors. The piezoelectric and piezoresistive effects of InAs NWs [116] were studied adopting *in situ* SEM tensile test method, as shown in Figure 5(d). The superior piezoelectric properties and piezoresistance effect of Sb-doped ZnO nanobelts were investigated through large longitudinal electromechanical characterization [117], as shown in Figure 5(e). In addition, the piezoresistive response of quasi-1D ZnO nanowires was characterized using an *in situ* SEM-based indentation system [118].

#### 2.3.3. MEMS-Based *In Situ* Electromechanical Characterization in SEM

The capability of simultaneous electrical and mechanical measurements of individual nanostructure has demonstrated MEMS devices as a popular platform for piezoresistivity characterization of single nanowires [79, 103, 119] and nanofiber [105] for the development of novel nanomechanical sensors. A MEMS device was developed for electromechanical characterization of nanowires [79]. Typically, individual nanowires are grown directly between actuators of MEMS device, so that the uniaxial tensile load can be applied to single NWs; for instance, carbon nanowires (CNWs) were fabricated on electrostatically actuated nanotensile tester by FIB-CVD [103]. Cu and SiC nanowires have been integrated into the MEMS tensile testing chip [120], and their mechanical and electronic properties under different stress were characterized simultaneously, as shown in Figure 5(f).

SEM could also be employed as crucial assistance for electromechanical characterization. In the investigation of structure dependent properties of carbon nanomaterials [121], the detailed packing structure and morphology have been revealed through SEM for the following electromechanical measurements. The effect of the morphology (checked by SEM) of the reduced graphene oxide (rGO) powders was studied through electromechanical measurements [122]. The electromechanical properties of the developed nanocomposite-based strain sensors are characterized in detail with the support of the cross-sectional SEM images of the packaged 1D sensing material [123]. In the direct electromechanical characterization of pressure-dependent contact current [124], the growth analysis of semiconductor NWs was examined using SEM. The characterization of piezoresistive polyurethane/graphene nanoplatelets coating for strain sensing applications was enabled by the morphological investigations of the produced nanomaterials using FE-SEM [125].

## 3. Perspective: Challenges and Future Work

### 3.1. Current Status: Few Optical-Related Characterization in SEM

With the rapid advance of optoelectronic devices, the optical and optoelectronic characterization of nanostructures is becoming more and more popular, which benefits the improvement of optoelectronic device as well as the determination of the correlation between properties and the geometrical parameters of nanostructures. However, as an important characterization platform, SEM was not often utilized for pure optical or optoelectronic characterization of nanomaterials, which is partially due to the technical challenge of integrating optical components inside the space-limited SEM chamber and achieving high-efficient optical excitation and measurement in the SEM environment. In the following texts, we will give a short review of optical-measurement-related characterization of nanomaterials in SEM and analyze the existing challenges for performing optoelectronic characterization in SEM.

For optical characterization of individual nanostructures, techniques of microphotoluminescence (micro-PL) [126] and scanning near field optical microscopy [126] were often employed in ambient environment. While in SEM, only the cathodoluminescence (CL) characterization of GaN film [127], GaN crystal [128], and ZnO columns [129] was reported previously. Also, the photoluminescence (PL) and CL characteristics of ZnO nanorod [130] were obtained by customized *in situ* optical characterization system in SEM. Some optoelectronic characterizations were assisted by focused ion beam (FIB) for deposition of metal contacts in an SEM chamber. For instance, the photoconductivity of ZnO NW-based UV photodetectors [131] was characterized with FIB-Pt deposition for electrical contacts.

### 3.2. Challenges for Optoelectronic Characterization in SEM

#### 3.2.1. Contact Resistance

In optoelectronic characterization of nanomaterials, the contact resistance between an electrode/nanoprobe and a sample could significantly affect the measured current-voltage (I-V) data; therefore, it is highly desired to minimize the contact resistance during electrical characterization of nanomaterials. Several studies have been reported for reducing the contact resistance of metal electrodes (formed by EBL or EBID) through rapid thermal annealing [132], electric current flowing [133], e-beam irradiation [134], and EBID [135].

#### 3.2.2. Contact Force/Pressure

During the process of *in situ* electrical characterization, applying a gentle contact force may ensure a good electrical contact; however, high applied contact forces/pressures pose risks of modifying the electrical properties of tested materials. Chen et al. [136] have investigated the effect of high contact forces/pressures on the modification of electrical properties using scalpel AFM and proposed a process flow to correct the use of scalpel AFM to characterize the electrical properties of nanomaterials in three dimensions.

#### 3.2.3. Efficient Light Detection in Limited-Size SEM Chamber

To perform optical characterization in SEM, efficient light collection and detection are necessary. A sizeable paraboloidal mirror was employed in a typical modern setup [137] for light collection in CL test; however, due to the limited space in the SEM chamber, the mirror blocked most other detectors and the electrical nanoprobe integration, thus hindered the simultaneous measurement of electrical and optical properties of nanodevices and nanomaterials. To address this issue, space-saving optical fibers [130] were integrated into the SEM chamber for *in situ* comprehensive optical characterization of individual optoelectronic nanostructures, providing inspiration for our strategy of optical fiber integration in SEM for optoelectronic characterization.

### 3.3. Future Work: Demand of Multiphysical Characterization System in SEM

Functional nanomaterials usually have unique multiphysical properties (mechanical, electrical, and optical properties) compared with their bulk counterparts. These multiphysical properties not only exist in independent states but also often coupled with each other and closed correlated as piezoelectric, photoplastic, and optoelectronic properties. For instance, widely studied multifunctional ZnO NWs possess unique physical characteristics such as semiconductivity and piezoelectricity and have found novel applications in sensors and biomedical science [138]. The observed remarkable photo-induced elastic effect in 1D semiconducting ZnO nanobelt [139] has demonstrated the significance of mechanical, optical, and electronic coupling in 1D nanostructures. Besides, III-nitride NWs (e.g., InN, GaN, and AlN) have been pivotal for optoelectronic applications such as ultrahigh-speed nanoscale lasers and photodetectors, full solar spectrum photovoltaic devices, and high-efficiency white light-emitting diodes (LEDs) [140]. These applications usually require optoelectronic characterization of the III-nitride NWs.

Therefore, for abovementioned nanomaterials, a SEM-based system capable of characterizing the mechanical, electrical, or optical property of nanomaterials independently or simultaneously is highly demanded. SEM-based *in situ* nanomanipulation technique owns several merits such as ultrahigh positioning resolution, accurately quantified mechanical excitation, and measurement on single nanostructure, as well as the nondestructive electrical nanoprobing capability of nanomaterials; all these merits will contribute to the establishment of SEM-based multiphysical characterization system of nanomaterials.

## 4. Conclusion

This review summarizes existing SEM-based platforms and SEM-enabled techniques for multiphysical (mechanical, electrical, and electromechanical) *in situ* characterization of nanomaterials. Also, a short review and existing challenges of optical-measurement-related characterization of nanomaterials in SEM are presented. From our perspectives, an SEM-based *in situ* multiphysical characterization system capable of characterizing the mechanical, electrical, or optical property of nanomaterials is in urgent need, which allows *in situ* assembly and comprehensive coupled-field nanomaterial characterization (optical, electrical, and mechanical) of individual nanomaterials and enables the systematic investigation of the complicated underlying coupled-field properties of advanced nanomaterials.

## Figures and Tables

**(a):** 

Testing types	Nanomaterials	Properties	References
Bending	MCNTs	Young's modulus	[29]

Buckling	MCNTs	Young's modulus	[18, 30]

Tensile	MCNTs	Strength and breaking mechanism	[31]
	InGaAs/GaAs nanosprings	Stiffness	[32]
	Si nanowires	Yield strength	[33, 34]
	Ag nanowires	Yield, ultimate tensile strength	[35]

Nanoindentation	Graphene membrane	Elastic stiffness and Young's modulus	[36]
	Graphene flakes	Young's modulus	[37]
	Nanopaper	Young's modulus	[38]
	InP membranes	Local stiffness	[39]
	CNF/LDPE nanocomposite	Surface mechanical properties	[40]

**Table:** (b) ESEM for biological cells

Nanomaterials	Properties	End-effectors	References
Wild type yeast cells	Single cell adhesion force	Nanofork	[46, 47]
	Cell-cell adhesion force	Nanopicker	[48]
	Cell-surface adhesion force	Flat AFM cantilever tips	[49, 50]
	Stiffness, viscoelastic properties	Soft buckling nanoneedles	[51–55]

Microbead and biological cell	Cell detachment force	FIB etched AFM cantilever	[56]

**Table 2:** MEMS-based mechanical characterization of micro- and nanomaterials.

Testing types	Materials	End-effectors	References
Tensile	2D thin films and beams		
	Pt films	Thermal actuator	[75]
	Freestanding Al films	Electrostatic actuator	[62, 64]
		Bent-beam thermal actuators	[65]
	Freestanding PolySi film	Thermal actuator	[59]
	Nanocrystalline Ni nanobeam	Thermal actuator	[58]
	CNTs		
	MCNTs	Thermal actuator	[59, 69]
		Electrostatic actuator	[63, 70]
	T-CNT	Thermal expansion beams	[68]
	1D nanowires		
	Ni nanowires	Nanoindenter head	[66]
	Carbon nanowires	Electrostatic actuator	[67]
	Co nanowires	Electrostatic actuator	[71]
	Si nanowires	Electrostatic actuator	[72]
	ZnO nanowires	Electrostatic actuator	[73]
	GaN nanowires	Thermal actuator	[74]

Bending	Polyacrylonitrile nanofibers	Folded-beam loadcell	[76]
	Microcantilever aluminum beam	Comb-drive probe	[62]
	Thin-film polysilicon microstructures	Rotary comb-drive actuator	[77]

**(a):** 

Testing types	Nanomaterials	Properties	References
Four-point probing	Boron nanowires	Conductivity	[80]
	CNTs	Current-voltage characteristics	[81–83]
	Graphene flake	Conductance	[84]
	SnO_2_ nanowires	Current-voltage characteristics	[85]

Two-point probing	InN nanowires	Electrical transport properties	[86]
	MCNTs	Current-voltage characteristics	[87, 88]
	GdSi_2_ nanowires	Electrical transport properties	[89]
	Bi_2_S_3_ nanowires	Current-voltage characteristics	[90]
	2D MXene nanosheets	Electrical conductivity	[91]

Three-point probing	CNT	Field effect measurements	[88]
	GdSi_2_ nanowires	Electrical conductance property	[92]
	Nb_2_Se_9_ FET	Electrical transport property	[93]

**Table:** (b) ESEM for biological cells

Nanomaterials	Properties	End-effectors	References
W303 yeast cells	Single cell electrical conductivity	Dual nanoprobes	[94]
W303 yeast cells	Single pulse current measurement	ESEM nanomanipulator system	[95]
Human embryonic kidney cell	Current response to indentation force	Robot-assisted AFM manipulation system	[96]

## Data Availability

The datasets used or reported in the current work are available from the corresponding author on reasonable request.
